# Plasma levels of TNF-α, IFN-γ, IL-4 and IL-10 during a course of experimental contagious bovine pleuropneumonia

**DOI:** 10.1186/1746-6148-8-44

**Published:** 2012-04-25

**Authors:** Flavio Sacchini, Mirella Luciani, Romolo Salini, Massimo Scacchia, Attilio Pini, Rossella Lelli, Jan Naessens, Jane Poole, Joerg Jores

**Affiliations:** 1 Istituto "G. Caporale", via Campo Boario, 64100 Teramo, Italy; 2 International Livestock Research Institute, Old Naivasha Road, PO Box 30709, 00100 Nairobi, Kenya

**Keywords:** Contagious bovine pleuropneumonia, *Mycoplasma mycoides *subsp. *mycoides*, Cytokines, TNF-α, IFN-γ, IL-4, IL-10

## Abstract

**Background:**

Contagious Bovine Pleuropneumonia (CBPP), caused by *Mycoplasma mycoides *subsp. *mycoides*, is widespread in sub-Saharan Africa. The current live vaccine T1/44 has limited efficacy and occasionally leads to severe side effects in the animals. A better understanding of the immune responses triggered by *Mycoplasma mycoides *subsp. *mycoides *and their role in disease progression will help to facilitate the design of a rational vaccine. Currently, knowledge of cytokines involved in immunity and immunopathology in CBPP is rather limited. The aim of this study was to characterize the *in vivo *plasma concentrations of the cytokines TNF-α, IFN-γ, IL-4, IL-10 and the overall role of CD4^+ ^T cells in the development of cytokine levels during a primary infection. Plasma cytokine concentrations in two groups of cattle (CD4^+ ^T cell-depleted and non-depleted cattle) experimentally infected with *Mycoplasma mycoides *subsp. *mycoides *were measured and their relationship to the clinical outcomes was investigated.

**Results:**

Plasma cytokine concentrations varied between animals in each group. Depletion of CD4^+ ^T cells did not induce significant changes in plasma levels of TNF-α, IL-4, and IL-10, suggesting a minor role of CD4^+ ^T cells in regulation or production of the three cytokines during the time window of depletion (1-2 weeks post depletion). Unexpectedly, the IFN-γ concentrations were slightly, but statistically significantly higher in the depleted group (p < 0.05) between week three and four post infection. Three CD4^+ ^T cell-depleted animals that experienced severe disease, had high levels of TNF-α and IFN-γ. Only one severely diseased non-depleted animal showed a high serum concentration of IL-4 post infection.

**Conclusions:**

Comparison of most severely diseased animals, which had to be euthanized prior to the expected date, versus less severe diseased animals, irrespective of the depletion status, suggested that high TNF-α levels are correlated with more severe pathology in concomitance with high IFN-γ levels.

## Background

Contagious bovine pleuropneumonia (CBPP), caused by *Mycoplasma mycoides *subsp. *mycoides*, is characterized by a severe fibrinous exudative pleuropneumonia. CBPP causes decreased productivity and direct losses of cattle, and on CBPP affected countries rigorous limitation to international trade are imposed in accordance with World Organization of Animal Health (OIE) regulation. The disease has been eradicated in Europe, Asia and America through the application of restrictions to the movement of cattle, as well as test and slaughter policies combined with compensation for livestock keepers. Such policies are difficult to apply in most African countries because of pastoralism, lack of economical resources, and fragmented veterinary services.

The current live vaccine, based on the attenuated strain T1/44, confers limited efficacy although it has been reported to have a degree of pathogenicity [1,2]. Annual revaccinations are necessary to confer a sufficient level of protection for the cattle population. An improved vaccine conferring long-term immunity is desirable for control of CBPP in Africa.

A comprehensive understanding of host pathogen interactions and the identification of protective versus counter protective immune responses are a prerequisite for the development of a rational vaccine. Currently, there is no clear understanding on how to induce solid immunity against *Mycoplasma mycoides *subsp. *mycoides *or what the main mechanisms of immunity are. Recent studies have focussed on antibody-mediated immunity [3,4] and T cell-related immunity [5-7]. However, conclusive results regarding protective or pathological immune responses could not be obtained. The immune response to infectious pathogens is mediated by cytokines, thus an understanding of the kinetics of the different cytokines in the course of disease is helpful in identifying correlates of both mild and severe CBPP. Different *Mycoplasma mycoides *subsp. *mycoides *strains activated *in vitro *bovine macrophages and induced the release of tumor necrosis factor alpha (TNF-α) [8]. Likewise heat-killed suspensions of the closely related pathogen *Mycoplasma mycoides *subsp. *capri *activated *in vitro *murine macrophages as well as bone marrow cells and induced cytokines such as TNF-α, ΙL-1, IL-6, and nitric oxide [9].

In this study the plasma levels of three proinflammatory (TNF-α, IFN-γ, IL-4) and one anti-inflammatory cytokine (IL-10) during a primary infection with *Mycoplasma mycoides *subsp. *mycoides *in ten temporarily CD4^+ ^T cell-depleted and ten non-depleted animals were measured, in order to correlate the plasma cytokine levels with disease outcome and additionally to estimate the role of CD4^+ ^T cells with respect to cytokine production.

## Methods

### Sample collection

All protocols of this study were designed and performed in strict accordance with the Kenyan legislation for animal experimentation and were approved by the institutional animal care and use committee (IACUC reference number 2008.08). Since 1993, ILRI has complied voluntarily with the United Kingdom's Animals (Scientific Procedures) Act 1986 that contains guidelines and codes of practice for the housing and care of animals used in scientific procedures.

The sample population of cattle experimentally infected with *Mycoplasma mycoides *subsp. *mycoides *has been described previously [5]. Briefly, 20 Kenyan Boran bullocks (*Bos indicus*) between 14 and 16 months of age were intra-tracheally infected using *Mycoplasma mycoides *subsp. *mycoides *Afadé. Ten cattle were depleted from CD4^+ ^T lymphocytes using a BoCD4 specific murine monoclonal antibody 6 days post infection (dpi). Cattle were kept for up to 30 dpi, euthanized and subjected to post mortem analysis. Blood samples were obtained before infection and twice a week post infection. Clinical outcome and pathomorphological lesions of the sample population have been described in detail before [5].

### Qualitative measurement of IgM, IgG1, IgG2 and IgA antibody levels

*Mycoplasma mycoides *subsp. *mycoides *Afadé was cultivated in liquid PPLO media at 37°C for 72 hours and log-phase culture centrifuged at 14000 × g at 4°C for 40 min. The cell pellet was washed three times with phosphate buffered saline (PBS) and resuspended in the same buffer. Protein concentration was determined using BCA protein assay kit (Thermo Scientific, Rockford, USA) according to the manufacturer instructions. Cells were diluted in bicarbonate buffer (0.05 M, pH 9.6), adjusting antigen concentration to 10 μg/ml.

ImmunoPolysorp 96-well standard plates (NUNC A/S Roskilde, Denmark) were coated with 100 μl/well of *Mycoplasma mycoides *subsp. *mycoides *Afadé total antigen as described above. Microplates were stored at 4°C overnight and washed with PBST buffer (0.01 M PBS, pH 7.2 with 0.05% Tween 20,). Each well was blocked against unspecific binding using 200 μl blocking buffer (PBST containing 1% of yeast extract) and microplates were incubated at 37°C for 1 hour and washed afterwards with PBST. Prediluted plasma samples (100 μl/well) were added for determination of IgA, IgG1, IgG2, and IgM responses. Plasma samples for IgM determination were prediluted 1:2560 while for the other ELISAs a 1:80 predilution was used. The samples were diluted in PBST with 0.1% of yeast extract. After incubation at 37°C for 1 hour microplates were washed using PBST, followed by incubation with secondary antibodies (100 μl/well) at room temperature for 1 hour. Antibody stock solutions were diluted in PBST with 0.1% of yeast extract, as outlined: murine anti-bovine IgG1 HRP conjugated (Abcam, Cambridge, UK), 1:150; murine anti-bovine IgG2 HRP conjugated (Abcam, Cambridge, UK), 1:150, murine anti-bovine IgA HRP conjugated (Bethyl Laboratories, Inc, Montgomery, USA), 1:800; murine anti-bovine IgM HRP conjugated (Bethyl Laboratories, Inc, Montgomery, USA), 1:1000. Afterwards the plates were washed and 100 μl/well of 3,3',5,5'-tetramethylbenzidine substrate was added, microplates were incubated at room temperature until optimal colour development was observed (5 minutes for IgM ELISA, 20 minutes for the other ELISAs) and reaction was stopped using 50 μl of 0.5 N sulphuric acid. Optical density (OD) was measured using a Benchmark microplate reader (Bio-Rad Laboratories) at a wavelength of 450 nm. All measurements have been carried out in duplicate.

### *In vivo *cytokine measurement

Blood samples were collected in Lithium Heparin tubes before and after experimental infection. Immediately after collection the samples were centrifuged at 4°C at 2000 × g for 15 min and the plasma was harvested and stored at -80°C for subsequent analysis. The concentrations of TNF-α, IFN-γ, IL-4 and IL-10 were assessed with the following commercially available ELISA kits: Bovine TNF-α duo set (R&D System Europe Ltd., UK); Bovine IFN-γ (Mabtech AB, Sweden); Bovine IL-4 Screening Set (Thermo Scientific, Pierce Biotechnology USA) and Bovine Interleukin 10 (Cusabio Biotech Co., LTD.). Individual tests were performed according to manufacturer's instructions. All samples were run in duplicate and results calculated by interpolation of OD sample values with the relevant standard curve using SigmaPlot^® ^11 Systat Software, Inc (San Jose, CA, USA) and expressed as protein concentration (pg/ml).

### Statistical analysis

Absolute cytokine concentrations were natural log transformed (TNF-α and IFN-γ) or square rooted (IL-10) before analysis, to satisfy normality assumptions. A two-sided two- sample t-test was used to compare the two groups at each time point. Prior to conducting the t-test the variances for the two groups were compared using an F-test. If the variances were significantly unequal then separate variances were used in the t-test, where the variances were not significantly different a pooled variance was used. Differences were considered statistically significant for p < 0.05.

## Results

### Sample population

All animals were successfully infected as shown by mounted antibody titres post infection and the isolation of the infectious agent from all animals [5]. The depletion of CD4^+ ^T cells was successful as demonstrated by flow cytometry [5]. Clinical symptoms of CBPP peaked between 12 and 15 dpi. Four animals, three from the depleted group (BD91, BD98, and BD118) and one from the non-depleted group (BD97), showed severe clinical symptoms and had to be euthanized before the planned end of the experiment for ethical reasons [5]. Cytokine levels of the four animals were compared with the 16 remaining animals, which had a milder form of clinical disease. The body temperature of the four animals was significantly higher than in the other animals (see Additional file 1: Comparison of body temperatures of cattle showing acute and mild disease symptoms). At post mortem examination, typical CBPP lesions were observed in 19 out of 20 animals [5]. Typology, extension and lesion severity were variable among the animals within the groups; no obvious differences, except the number of animals, which succumbed to disease, between CD4^+ ^ T cell-depleted and non-depleted groups were observed [5].

### Observed cytokine levels during experimental infection in non-depleted animals

**Figure 1:**
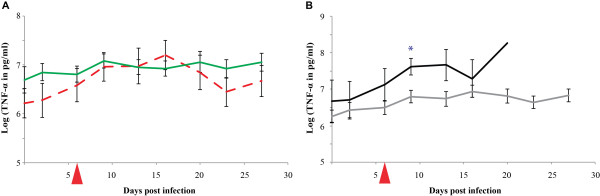
**TNF-α plasma levels during an experimental infection with *Mycoplasma mycoides *subsp. *mycoides *Afadé**. Comparison of TNF-α plasma levels in CD4^+ ^T cell-depleted animals (dashed red line) and non-depleted animals (green line) is shown in Figure 1A while the comparison of plasma levels in severe (black line) and chronic disease (grey line) outcome is displayed in Figure 1B. The day of depletion of CD4^+ ^T cells is indicated by a red triangle. The standard error bars are displayed. Significant levels are marked (* = p < 0.05).

The average TNF-α plasma levels increased slightly after experimental infection and ranged from 1100 pg/ml to > 1400 pg/ml (see Figure 1A and Additional file 2: TNF-α plasma concentrations in pg/ml). However, one animal (BD106) showed a high TNF-α plasma concentration before infection. In this animal the measured plasma concentration of 5400 pg/ml before infection dropped to 2000-3000 pg/ml after experimental infection, despite the fact that its health status was good at the beginning of the experiment.

**Figure 2:**
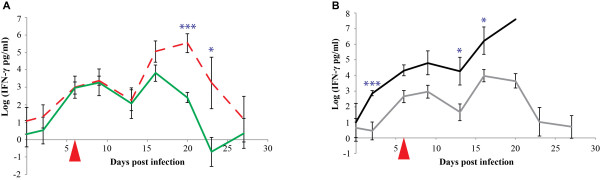
**IFN-γ plasma levels during an experimental infection with *Mycoplasma mycoides *subsp. *mycoides *Afadé**. Comparison of IFN-γ plasma levels in CD4^+ ^T cell- depleted animals (dashed red line) and non-depleted animals (green line) is shown in Figure 2A while the comparison of plasma levels in severe (black line) and chronic disease (grey line) outcome is displayed in Figure 2B. The day of depletion of CD4^+ ^T cells is indicated by a red triangle. The standard error bars are displayed. Significant levels are marked (* = p < 0.05, ** = p < 0.01, *** = p < 0.001).

The average IFN-γ plasma concentrations increased post infection from 6.5 pg/ml to > 100 pg/ml and then remained constant for about two weeks before dropping three weeks post infection to pre-infection levels (see Figure 2A and Additional file 3: IFN-γ plasma concentrations in pg/ml). The concentration of IFN-γ peaked as high as 1140 pg/ml 9 dpi in animal BD97, which showed severe clinical and patho-morphological signatures.

IL-4 was only detected in this one severely affected animal, BD97. The IL-4 concentration increased post infection from zero up to 340 pg/ml before dropping to 100 pg/ml before euthanasia. Plasma levels of IL-4 in all other animals remained below the assay detection limit (see Additional file 4: IL-4 plasma concentrations in pg/ml).

**Figure 3:**
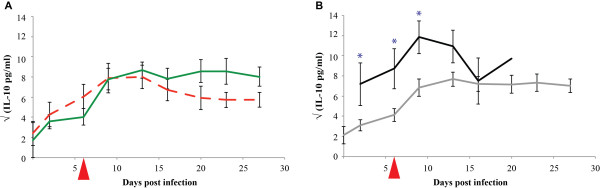
**IL-10 plasma levels during an experimental infection with *Mycoplasma mycoides *subsp. *mycoides *Afadé**. Comparison of IL-10 plasma levels in CD4^+ ^T cell-depleted animals (dashed red line) and non-depleted animals (green line) is shown in Figure 3A while the comparison of plasma levels in severe (black line) and chronic disease (grey line) outcome is displayed in Figure 3B. The day of depletion of CD4^+ ^T cells is indicated by a red triangle. The standard error bars are displayed. Significant levels are marked (* = p < 0.05).

The average IL-10 plasma levels increased post infection from 10 pg/ml to 80 pg/ml and stayed high throughout the experiment (see Figure 3A and Additional file 5: IL-10 plasma concentrations in pg/ml). The highest concentrations were observed in animal BD115, which remained above 200 pg/ml for most of the time; the peak level was nearly 300 pg/ml.

### Comparison of plasma cytokine levels in CD4^+ ^T cell-depleted and non-depleted cattle

In order to determine whether CD4^+ ^T cell-depleted cattle had a different cytokine plasma profile we compared the two groups (CD4^+ ^T cell-depleted and non-depleted) of 10 cattle each using their cytokine plasma concentrations and tested for significant differences.

### TNF-α

The average TNF-α plasma levels increased in both groups post infection. The average levels peaked around 1 - 2 weeks post infection and afterwards dropped close to the initial level in the depleted group while remained higher than the initial levels in the non- depleted group (Figure 1A). The differences observed were not statistically significant at the 5% level when compared to the results of the non-depleted group.

### IFN-γ

The average IFN-γ plasma levels of the CD4^+ ^T cell-depleted group increased in synchrony with the levels of the non-depleted group up to three weeks post infection. The levels dropped afterwards to the values before infection. The IFN-γ plasma levels measured from 20 dpi up to 23 dpi were significantly higher in the CD4^+ ^T cell-depleted group (p < 0.001 and p = 0.029, respectively) (Figure 2A).

### IL-4

None of the CD4^+ ^T cell-depleted animals showed IL-4 levels above the detection limit, while only one non-depleted animal (BD97) showed an increased level, which did not allow a statistical comparison of the two groups.

### IL-10

The average IL-10 plasma levels of the CD4^+ ^T cell-depleted group increased after infection as in the non-depleted group. The average levels peaked around 1-2 weeks post infection and then dropped slightly. The differences observed were not statistically significant at the 5% level when compared to the results of the non-depleted group.

### Comparison of plasma cytokine levels in animals with severe clinical disease and animals with a milder clinical form of disease

In order to assess the presence and role of cytokines with respect to the outcome of an infection with *Mycoplasma mycoides *subsp. *mycoides *the animals were grouped according to their clinical disease severity (severe disease patterns versus milder disease patterns) and the cytokine plasma profiles for the two groups were compared. The four animals BD91, BD97, BD98, and BD118 showed the most severe course of disease and were euthanized before the planned end of the experiment. Their pathomorphological outcome correlated with high disease severity. Therefore, these four animals were placed into the severe disease group and statistically compared to the remaining animals (mild disease).

### TNF-α

The average TNF-α plasma levels in the severe disease group were higher than in the mild disease group throughout the experiment. The difference was statistically significant at 9 dpi (p = 0.031) and remained borderline different at 13 dpi (p = 0.057) (Figure 1B). The levels increased in all animals after infection.

### IFN-γ

The average IFN-γ plasma levels in the severe disease group was higher than in the mild disease group throughout the experiment. The difference was significant from 2 dpi (p < 0.001) through 16 dpi (p = 0.022) when the severe disease group of animals began being removed from the trial (Figure 2B).

### IL-4

Only one animal (BD97) in the severe disease group showed an increased level, which did not allow for statistical comparisons between the groups.

### IL-10

The average IL-10 plasma levels of both the mild and severe disease groups increased after infection. The average levels peaked around 1 - 2 weeks post infection and afterwards dropped slightly. The levels were always higher in the severe disease group and statistically significantly higher (p < 0.05) than the mild disease group in the first 10 dpi (Figure 3B).

### Comparison of plasma immunoglobulin responses in CD4^+ ^T cell-depleted and non-depleted cattle

In order to determine whether CD4^+ ^T cell-depleted cattle had a different antibody response we compared the two groups (CD4^+ ^T cell-depleted and non-depleted) using ELISA technique and tested for significant differences.

CD4^+ ^T cell-depleted cattle had a significant higher IgG1 and IgA titres after depletion at two time points (see Additional file 6: Observed qualitative Mycoplasma-specific antibody responses). IgG2 and IgM titres did not differ significantly between groups.

### Comparison of plasma immunoglobulin responses in animals with severe clinical disease and animals with a milder clinical form of disease

In order to determine whether animals with severe clinical disease and animals with a milder clinical form of disease had a different antibody response we compared the two groups using ELISA technique and tested for significant differences.

Animals with severe clinical disease had a significant higher IgG1 (3 time points), IgG2 (2 time points) IgA and IgM (only 1 time point) (see Additional file 6: Observed qualitative Mycoplasma-specific antibody responses).

## Discussion

Currently, protective and inflammatory responses during CBPP are not fully understood. In recent years, IFN-γ and especially IFN-γ-secreting CD4^+ ^T cells have been proposed as key elements required for protection [10], as their levels were inversely correlated with severity of disease. A recent study employing murine CD4^+ ^specific mAb to deplete bovine CD4^+ ^T cells, including the IFN-γ-secreting population, represents the first attempt to investigate, *in vivo*, the role of CD4^+ ^T cells in a primary CBPP infection [5]. Animals were depleted at 6 dpi, since the time window of complete depletion is less than two weeks and we wanted to time depletion in the onset of clinical disease. It cannot be ruled out that CD4^+ ^T cells contributed to CBPP-specific cytokine responses before depletion. Four out of twenty animals had to be euthanized before the envisaged end of the trial because of ethical reasons. This is in the range of reported mortality of CBPP especially given the fact that those animals were kept at ideal conditions with food *ad libitum *and no climate or production-associated stress. The animals were monitored for selected *in vivo *cytokine plasma profiles during *Mycoplasma mycoides *subsp. *mycoides *infection to see whether the depletion of most CD4^+ ^specific T cells over a time window had any effects on the cytokine profiles. Flow cytometry analysis had demonstrated that the CD4^+ ^T cell depletion was successful and that naïve CD4^+ ^T cells reappeared approximately ten days post depletion, but did not reach the levels of control animals until the end of the experiment [5].

Acute and sub-acute cases of CBPP are characterised by strong inflammatory reaction leading to serofibrinous pleuropneumonia, pleural effusion and tissue damage. We expected a correlation between increased TNF-α plasma levels and severe clinical signs and/or lesions, since TNF-α is one of the key inflammatory cytokines that it is released in large amounts following infection with gram-negative and other bacteria including mycoplasmas and is responsible for systemic complications [11]. An increase of TNF-α in the plasma was observed in most of the animals following infection, which supports the hypothesis that TNF-α mediates inflammation in CBPP. The levels did not increase dramatically, which might be attributed to the fact that TNF-α is produced locally, has a short half-life, and so primarily accomplishes its effects locally. Therefore, the increase of the plasma levels might be a signature of what is happening in certain regions of the lung.

The high degree of lung lobulation and the absence of collateral airways in the bovine lung often quarantine inflammatory processes to defined lobes [12] and prevent an inflammation of the entire lung as seen only in a small fraction of the animals after experimental infection. The most severely infected animals showed a significantly higher level of plasma TNF-α when compared to the animals showing a milder reaction. However, it must be noted that only three out of four animals most severely affected showed very high concentrations of the cytokine. Conversely, one animal with a mild form of disease had a very high plasma concentration of TNF-α throughout the experiment. The data further suggest that the CD4^+ ^T cell depletion did not have a significant effect on the TNF-α plasma levels. This is not surprising, as cells from the macrophage lineage, which are amply represented by alveolar macrophages, are important producers of TNF-α.

Although IFN-γ is known to prime macrophages to secrete more TNF-α after stimulation, the IFN-γ in this experiment frequently peaked later (BD 91, BD 93, BD 95, BD 98, BD 107, BD 118) rather than simultaneously with TNF-α. This time difference suggests that early TNF-α secretion is not highly dependent on plasma IFN-γ. Again, local IFN-γ release in the lungs, which could not be detected given the set-up of this experiment, might have different kinetics and affected TNF-α concentrations. It has been shown *in vitro *that direct exposure of bovine alveolar macrophages to *Mycoplasma mycoides *subsp. *mycoides *can trigger TNF-α secretion [8]. *In vivo *IFN-γ measurement showed increased release of the cytokine in plasma from the depleted group 16 dpi (ten days post CD4^+ ^T cell depletion). During this period the percentage of CD4^+ ^cells was less than 1% as demonstrated by flow cytometric analysis. The CD4^+ ^T cells that re-appeared derived most likely from the thymus and were naïve, thus not capable of mounting a prompt immune response [13]. This implies that CD4^+ ^T cells are not the main or most important source of IFN-γ in the course of CBPP, and that other cellular compartments must be responsible for IFN-γ production. Natural killer cells, CD8^+ ^T cells or γδ T cells are also capable of producing IFN-γ, but their role in CBPP immune response has not been elucidated.

Animals that had to be euthanized because of severe clinical signs had significantly higher levels of plasma IFN-γ in comparison to animals with low pathology scores. On the other hand, animals with low pathology scores did not show high IFN-γ responses.

This was surprising; since previous studies had shown that higher IFN-γ levels in stimulated cultures of peripheral blood mononuclear cells (PBMCs) correlated with milder disease [10]. Other studies based on similar *in vitro *cell culture assays did not confirm the positive correlation between resistance and IFN-γ recall responses from PBMCs [14]. While TNF-α and IFN-γ may have a protective effect or stimulate protective responses, their concentrations may be linked to the severity of the infection and the amount of mycoplasma in the host. Bovine IL-4 is not a Th2 signature cytokine but is associated to a Th2 profile [15].

Interestingly, measurable IL-4 plasma concentrations were only detected in one non-CD 4^+ ^T cell-depleted animal (BD97) that developed severe CBPP infection. This animal also presented high IFN-γ levels suggesting that acute and severe CBPP cases develop an imbalanced cytokine release with high level of IFN-γ and IL-4 resulting in severe pathology as reported in other cattle diseases and in some cases of human mycobacterial infection [16].

Analysis of the anti-inflammatory cytokine IL-10, which increased in most of the animals between 6 and 13 dpi, showed an interesting correlation between augmented release of IL-10 and increased plasma levels of TNF-α or IFN-γ. Since most of the animals showing increasing IL-10 concentrations had pathomorphological signatures of CBPP, we speculate that the increase in IL-10 is not sufficient to prevent excessive inflammatory processes that shape CBPP lung lesions. Future experiments should include the monitoring of this cytokine in immune animals. Our findings did not indicate a prominent role for CD4^+ ^T cells in IL-10 secretion and it is known that many populations of bovine cells, including Th0, Th1 and Th2 cells, can produce IL-10 [17]. Stimulated human alveolar macrophages can be induced to produce IL-10 [18], but similar data for cattle are missing.

We characterized IgG1, IgG2, IgA, and IgM antibody responses against Mycoplasma antigen post infection. As we did not evaluate the binding affinities of the secondary reagents we were not able to draw meaningful conclusions regarding a T1 or T2 bias based upon the ratio of IgG1 to IgG2 titres [15]. We observed statistically higher IgG1 and IgG2 responses (see Additional file 6: Observed qualitative Mycoplasma-specific antibody responses) in severely affected animals. Future studies should evaluate the biological role of these responses, in particular a possible involvement of immune complexes in disease progression. Immune complexes are likely to be formed during CBPP as a result of high levels of immune globulins in combination with proinflammatory cytokines. This is the first study looking for cytokine signatures in cattle that have been infected with *Mycoplasma mycoides *subsp. *mycoides*. Future studies should focus on whether the cytokine kinetics observed are the cause of pathoimmunological reactions or a manifestation of it in order to understand the disease, design proper control measures and direct the immune responses of next generation vaccines properly.

## Conclusion

Analysis of selected *in vivo *cytokine profiles during a course of CBPP showed a predominant IFN-γ-mediated T1 type of response (IFN-γ with no IL-4) in three out of four animals with most severe clinical course of disease and pathological lesions. The higher levels of IL-10, TNF-α and IFN-γ in animals showing the most severe clinical disease strongly suggest their involvement as either a driver or mediator of immune reactions towards the pathogen. A lack of CD4^+ ^T cells seems to enhance the level of plasma IFN-γ during the course of disease. According to our results we speculate that high levels of IFN-γ produced in the early stages of *Mycoplasma mycoides *subsp. *mycoides *infection is disadvantageous to the host. Additional studies are required to investigate in parallel the cytokine network that takes place in the lung following *Mycoplasma mycoides *subsp. *mycoides *infection.

## Abbreviations

CBPP: Contagious bovine pleuropneumonia; dpi: Days post infection; ELISA: Enzyme-linked Immunosorbent Assay; IFN-γ: Interferon gamma; IgA: Immunoglobulin A; IgG: Immunoglobulin G; IgM: Immunoglobulin M; IL-4: Interleukin 4; IL-10: Interleukin 10; OD: Optical density; TNF-α: Tumor necrosis factor alpha.

## Authors' contributions

FS and JJ designed the study. FS and JJ drafted the manuscript. FS and ML did the ELISA work. JP and RS did the statistical analysis. All authors interpreted the data and revised critically the manuscript. FS and JJ coordinated the whole study. All authors read and approved the final manuscript.

## Supplementary Material

Additional file 1 **Comparison of body temperatures of cattle showing acute and mild disease symptoms**. Two groups of animals, which showed acute clinical symptoms (red) and mild clinical symptoms (blue) are displayed. Significant levels are marked (* = p < 0.05, ** = p < 0.01, *** = p < 0.001). Click here for file

Additional file 2 **TNF-α plasma concentrations in pg/ml**. Click here for file

Additional file 3 **IFN-γ plasma concentrations in pg/ml**. Click here for file

Additional file 4 **IL-4 plasma concentrations in pg/ml**. Click here for file

Additional file 5 **IL-10 plasma concentrations in pg/ml**. Click here for file

Additional file 6 **Observed qualitative *Mycoplasma*-specific antibody responses**. The day of depletion of CD4^+ ^T cells is indicated by a red triangle. Click here for file
