# Supplementary material for: Plasma levels of TNF-α, IFN-γ, IL-4 and IL-10 during a course of experimental contagious bovine pleuropneumonia
Source: BMC Vet Res. 2012 Apr 25;8:44. doi: 10.1186/1746-6148-8-44 (PMC3378467; doi:10.1186/1746-6148-8-44)

Additional File 1: Comparison of body temperatures in cattle showing acute and milder clinical symptoms

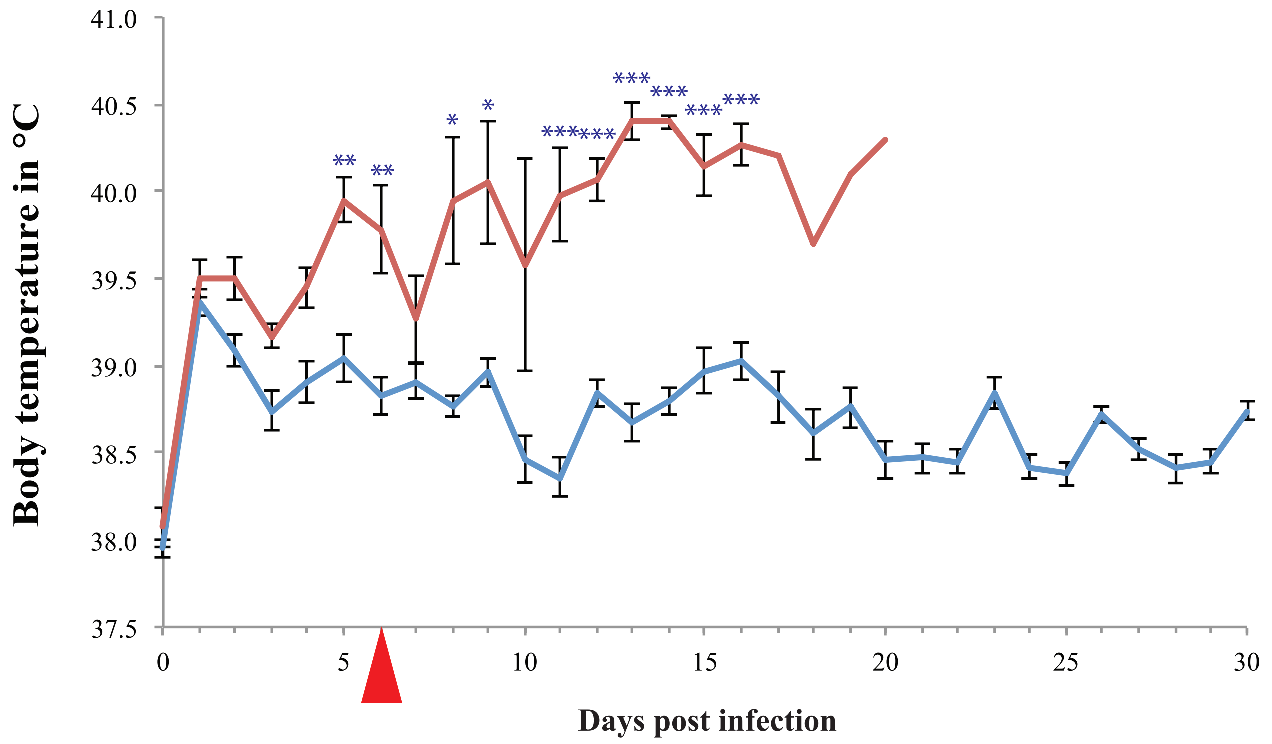

Supplement: Additional file 1 — Comparison of body temperatures of cattle showing acute and mild disease symptoms. Two groups of animals, which showed acute clinical symptoms (red) and mild clinical symptoms (blue) are displayed. Significant levels are marked (* = p < 0.05, ** = p < 0.01, *** = p < 0.001). [file 1746-6148-8-44-S1.PDF]
