# Supplementary material for: Plasma levels of TNF-α, IFN-γ, IL-4 and IL-10 during a course of experimental contagious bovine pleuropneumonia
Source: BMC Vet Res. 2012 Apr 25;8:44. doi: 10.1186/1746-6148-8-44 (PMC3378467; doi:10.1186/1746-6148-8-44)
Supplement: Additional file 2 — TNF-α plasma concentrations in pg/ml. [file 1746-6148-8-44-S2.PDF]

Additional File 2: TNF-α plasma concentrations in pg/ml

	Days p.i.	0	2	6	9	13	16	20	23	27
Animal number ($CD4^+$ T cell depleted animals are displayed in bold)	**BD91**	2125.6	2810.6	3697.9	2825.4	4142.3	3290.7			
	BD92	266.7	504.9	440.6	908.2	841.4	850.6	749.5	387.4	475.7
	**BD93**	1255.5	1736.1	4011.8	4178.5	4606.5	3648.7	3451.8	1868.9	2810.5
	**BD94**	219.4	376.4	202.3	738.7	425.0	607.7	356.6	232.4	200.5
	BD95	266.7	580.0	882.3	1429.5	2307.7	1853.2	3079.7	1383.9	1990.7
	**BD96**	398.7	501.3	727.2	821.9	763.7	2235.5	1066.2	800.3	815.3
	BD97	784.8	698.5	1191.0	1606.9	877.5	522.8			
	**BD98**	1506.1	994.5	1297.0	3080.4	4742.7	3910.4	3882.3		
	**BD99**	214.2	60.7	230.6	253.2	264.1		466.3	375.6	701.4
	**BD100**	295.6	730.8	487.0	540.1	658.6	904.4	784.2	911.8	1365.2
	**BD101**	992.3	792.0	1096.2	1233.7	1641.4	1164.9	1347.0	1192.3	1075.9
	BD102	1135.8	1172.7	947.4	1480.9	1432.3	1504.2	1607.3	1627.9	1477.3
	BD105	855.5	716.4	612.3	608.9	760.9	986.8	1449.6	1143.2	1127.9
	BD106	5440,4	2970,3	2935,2	3077,3	2094,9	2367,3	2729,6	2546,2	2612,7
	BD107	1102.6	1915.2	922.1	1180.1	944.4	1050.3	743.2	1008.9	1072.9
	BD111	882.8	969.1	1103.5	1182.9	1070.4	891.7	1085.3	1152.4	1094.2
	BD115	655.3	806.5	849.7	992.0	834.8	702.2	642.3	588.2	549.6
	BD116	615.9	748.8	548.4	697.2	475.7	620.3	522.8	656.6	1446.1
	**BD118**	156.2	223.5	414.1	1208.3	1190.4	689.6			
	**BD119**	287.5	251.9	280.4	384.0	209.7	312.8	249.9	279.1	407.9
