# Supplementary material for: Plasma levels of TNF-α, IFN-γ, IL-4 and IL-10 during a course of experimental contagious bovine pleuropneumonia
Source: BMC Vet Res. 2012 Apr 25;8:44. doi: 10.1186/1746-6148-8-44 (PMC3378467; doi:10.1186/1746-6148-8-44)
Supplement: Additional file 3 — IFN-γ plasma concentrations in pg/ml. [file 1746-6148-8-44-S3.PDF]

Additional File 3: IFN-γ plasma concentrations in pg/ml

	**Days p.i.**	**0**	**2**	**6**	**9**	**13**	**16**	**20**	**23**	**27**
**Animal number ($CD4^+$ T cell depleted animals are displayed in bold)**	**BD91**	11.3	17.0	36.5	89.1	51.0	1935.4			
	BD92	7.2	13.0	13.7	70.6	5.2	9.5	10.5	0.0	0.0
	**BD93**	40.2	26.2	61.3	62.0	13.3	1979.0	118.9	60.1	0.0
	**BD94**	20.5	0.0	10.9	13.4	18.0	190.5	183.3	91.9	103.5
	BD95	0.0	0.0	4.2	7.0	6.4	215.9	15.0	3.2	0.0
	**BD96**	11.6	7.0	41.1	13.7	38.0	123.7	238.5	298.2	0.0
	BD97	2.0	11.9	129.7	1143.6	813.5	360.2			
	**BD98**	0.0	16.1	48.1	47.1	60.5	47.4	1942.1		
	**BD99**	4.7	8.3	20.0	40.7	20.5	12.1	1663.4	1757.6	0.0
	**BD100**	3.7	0.0	0.0	16.8	0.0	17.7	296.3	263.2	17.0
	**BD101**	0.0	2.0	27.1	54.3	4.0	31.9	14.2	0.0	6.1
	BD102	24.2	17.8	5.5	27.4	3.8	33.6	9.2	0.0	12.1
	BD105	0.0	0.0	9.1	13.4	0.0	322.5	5.0	0.0	0.0
	BD106	11.2	0.0	51.7	0.0	0.0	34.5	2.2	0.0	0.0
	BD107	0.0	0.0	8.7	87.2	21.2	11.0	15.4	0.0	11.3
	BD111	15.8	11.0	55.0	92.6	57.1	38.4	48.6	10.5	18.0
	BD115	4.2	5.2	32.9	14.4	13.0	5.2	9.1	57.1	4.2
	BD116	0.0	11.6	22.1	63.5	65.2	54.5	22.0	0.0	22.7
	**BD118**	22.4	27.4	139.1	44.8	10.7	1966.2			
	**BD119**	0.0	2.5	28.0	6.2	2.5	152.4	229.1	0.0	323.1
