# Supplementary material for: Plasma levels of TNF-α, IFN-γ, IL-4 and IL-10 during a course of experimental contagious bovine pleuropneumonia
Source: BMC Vet Res. 2012 Apr 25;8:44. doi: 10.1186/1746-6148-8-44 (PMC3378467; doi:10.1186/1746-6148-8-44)
Supplement: Additional file 5 — IL-10 plasma concentrations in pg/ml. [file 1746-6148-8-44-S5.PDF]

Additional File 5: IL-10 plasma concentrations in pg/ml

	Days p.i.	0	2	6	9	13	16	20	23	27
Animal number ($CD4^+$ T cell depleted animals are displayed in bold)	**BD91**		84.4	96.1	225.3	228.0	196.3			
	BD92	11.9	18.0	9.9	64.3	68.9	59.3	71.9	60.7	63.0
	**BD93**		0.0	30.4	62.1	77.9	116.1	76.6	44.3	48.2
	**BD94**	13.3	8.8	21.4	57.2	81.1	44.2	47.8	56.5	47.1
	BD95		11.7	4.8	20.7	73.0	37.2	49.0	26.4	32.1
	**BD96**		9.3	10.3	48.7	29.0	30.4	35.4	39.5	61.3
	BD97		10.7	17.0	52.8	63.8	14.5			
	**BD98**		16.6	54.4	150.7	138.9	50.7	94.6		
	**BD99**		19.6	23.8	70.5	49.9	32.4	37.2	23.8	11.0
	**BD100**		3.9	12.4	18.7	17.5	18.4	4.8	16.5	20.4
	**BD101**	0.0	0.0	0.0	0.0	3.9	6.8	0.0	6.2	11.3
	BD102		0.0	4.1	20.6	19.0	17.4	18.2	19.1	30.3
	BD105	0.0	0.0	0.0	41.5	50.7	39.6	21.8	28.2	22.9
	BD106		10.8	11.0	45.5	67.3	79.6	90.3	85.8	80.2
	BD107		22.3	39.6	174.0	121.2	88.0	64.5	109.5	75.8
	BD111		12.0	15.4	43.7	48.8	42.7	81.5	67.7	67.1
	BD115		55.1	85.9	213.0	189.2	211.9	249.5	294.1	206.8
	BD116		33.8	35.4	34.1	107.4	117.6	107.4	86.3	65.8
	**BD118**		149.3	183.1	163.0	77.0	25.2			
	**BD119**	12.6	24.0	67.4	12.2	61.4	27.8	57.4	67.4	53.8
