# Supplementary material for: Plasma levels of TNF-α, IFN-γ, IL-4 and IL-10 during a course of experimental contagious bovine pleuropneumonia
Source: BMC Vet Res. 2012 Apr 25;8:44. doi: 10.1186/1746-6148-8-44 (PMC3378467; doi:10.1186/1746-6148-8-44)
Supplement: Additional file 6 — Observed qualitative Mycoplasma-specific antibody responses. The day of depletion of CD4+ T cells is indicated by a red triangle. [file 1746-6148-8-44-S6.PDF]

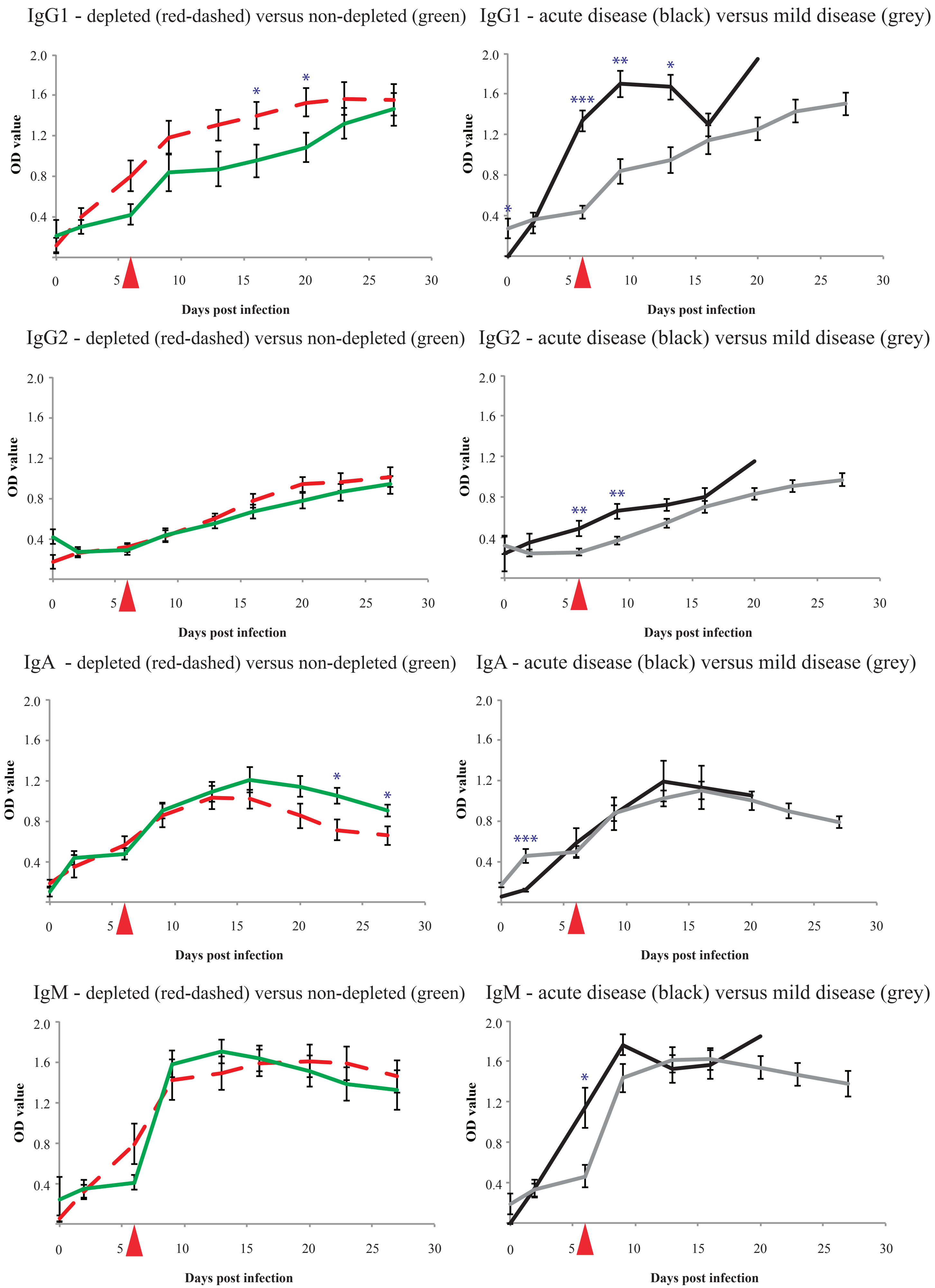
IgG1 - depleted (red-dashed) versus non-depleted (green)
IgG1 - acute disease (black) versus mild disease (grey)
IgG2 - depleted (red-dashed) versus non-depleted (green)
IgG2 - acute disease (black) versus mild disease (grey)
IgA - depleted (red-dashed) versus non-depleted (green)
IgA - acute disease (black) versus mild disease (grey)
IgM - depleted (red-dashed) versus non-depleted (green)
IgM - acute disease (black) versus mild disease (grey)
OD value
Days post infection
0.4
0.8
1.2
1.6
2.0
0
5
10
15
20
25
30
*
**
***
